# Differential item functioning of the SF-12 in a population-based regional joint replacement registry

**DOI:** 10.1186/s12955-019-1166-1

**Published:** 2019-07-02

**Authors:** Iraj Yadegari, Eric Bohm, Olawale F. Ayilara, Lixia Zhang, Richard Sawatzky, Tolulope T. Sajobi, Lisa M. Lix

**Affiliations:** 1 Department of Mathematics and Statistics, University of Ottawa, Ottawa, ON Canada; 2 Department of Community Health Sciences, University of Manitoba, S113-750 Bannatyne Avenue, Winnipeg, MB R3E 0W3 Canada; 3 Department of Surgery, University of Manitoba, Winnipeg, MB Canada; 4 School of Nursing, Trinity Western University, Langley, BC Canada; 5 Department of Community Health Sciences, University of Calgary, Calgary, AB Canada

**Keywords:** Arthroplasty, Clinical registry, Health-related quality of life, Measurement bias

## Abstract

**Background:**

Joint replacement, an increasingly common procedure amongst older adults, can substantially improve health-related quality of life (HRQoL). However, differential item functioning (DIF) may affect the accurate interpretation of differences in HRQoL amongst patients with different demographic and health status characteristics but the same underlying (i.e., latent) level of the investigated construct. This study tested for DIF in pre-operative SF-12 physical health (PH) and mental health (MH) sub-scale items amongst patients undergoing total hip arthroplasty (THA) and total knee arthroplasty (TKA).

**Methods:**

Data were from a population-based joint replacement registry from the Canadian province of Manitoba. TKA and THA patients who had surgery between 2009 and 2015 and completed a pre-operative assessment were included. DIF was tested using the multiple indicators multiple causes (MIMIC) method with sex, age group, body weight status, and presence of multiple comorbid conditions (i.e., multimorbidity) as covariates. Analyses were stratified by joint type.

**Results:**

The study cohort included 8820 patients; 42.1% underwent THA, 57.3% were female, 32.7% were 70+ years, and 52.8% were obese. For each sub-scale, four of the six items exhibited DIF in both THA and TKA groups. Differences in the covariate effect estimates for DIF and No-DIF models on the MH latent variable were largest for age and body weight status for the THA group, and for sex and multimorbidity for the TKA group. All of the differences were small for PH. Multimorbidity had the strongest association with PH and age and sex had the strongest association with MH in the DIF models.

**Conclusions:**

Demographic and health status characteristics influenced SF-12 PH and MH item responses in joint replacement populations, although the size of the effects were not large for PH. We recommend testing and adjusting for DIF effects to ensure comparability of HRQoL measures in joint replacement populations.

**Electronic supplementary material:**

The online version of this article (10.1186/s12955-019-1166-1) contains supplementary material, which is available to authorized users.

## Background

Joint replacement is an increasingly common procedure; rates of total hip and knee arthroplasty (THA/TKA) are increasing worldwide [1, 2]. THA and TKA can positively impact the health-related quality of life (HRQoL) of patients, resulting in substantial improvements in functional abilities and reductions in pain [3, 4]. There is strong interest worldwide in the incorporation of patient-reported outcome measures (PROMs) into joint replacement registries for monitoring appropriateness of care, improvements in health status, and health system performance [5]. The International Society of Arthroplasty Registries has convened working groups to evaluate and advise on best practices in the selection, administration, and interpretation of PROMs for joint replacement registries [6, 7].

Measurement validity and reliability are key considerations in the interpretation of patient responses on PROMs. An important validity criterion relevant to group comparisons is that the scoring of PROMs must be free from the effects of differential item functioning (DIF), which arises when patients with the same underlying level of the latent trait that the PROM is intended to measure do not interpret a PROM’s items in the same way [8]. DIF results in different item response probabilities for individuals with similar observed characteristics [9]. If DIF is present, then observed group differences will at least partially reflect something other than the latent construct, such as different interpretations of the item(s). DIF can result in biased between-group comparisons because the response patterns may reflect attributes other than those that the instrument is intended to measure.

Brief general-purpose PROMs, such as the 12-item Short Form Survey (i.e., SF-12), are advantageous to administer to joint replacement patients because they facilitate comparisons across patient populations while reducing participant response burden at pre- and post-operative measurement occasions. The SF-12 has undergone comprehensive psychometric evaluations of its reliability and validity [10].

Although DIF has been tested in other measurement instruments [9, 11–13], only a few studies have investigated DIF for the SF-12. DIF has been detected for the SF-12 in population-based data [14, 15]; a study in the general population revealed DIF effects by age, sex, and level of education [14]. However, DIF has not been thoroughly investigated in specific populations, such as in joint replacement populations.

The goal of our study was to test for DIF on the SF-12 physical health (PH) and mental health (MH) sub-scale items in a joint replacement population. We considered demographic characteristics in addition to health status characteristics in assessing DIF; the latter have recently been examined as potential contributors to DIF in PROMs for patients with osteoarthritis [16] and joint pain [17].

## Methods

### Data source

Data were from the Winnipeg Regional Health Authority Joint Replacement Registry; the Health Authority is the largest health region in the province of Manitoba, Canada and has a population of more than 700,000 residents. The province has a single-payer health care system that provides necessary hospital, medical and surgical services to all individuals eligible to receive health services. The Registry captures more than 90% of the joint replacement procedures conducted within the health region and more than three-quarters of all procedures in the entire province.

The Registry was initiated in 2004 with partial capture of information on all joint replacement surgeries; this was expanded to full mandatory capture of information in 2005. The Registry has been described in detail elsewhere [18]; it contains patient demographics, comorbid conditions, surgical technique, implant details, and complications. Both general and condition-specific HRQoL measures are included in the Registry. The former includes the SF-12 and the latter includes the Oxford Hip and Knee scores [19, 20]. Pre-operative data capture occurs in the pre-admission clinic under the guidance of a clinic nurse. Post-operative data are collected via a mail-out questionnaire conducted by Registry staff. Data entry is undertaken by the hospital medical records department for hospital stay characteristics and by Registry staff for PROMs. All data are collected via standardized instruments and the process of data collection and entry is overseen by Registry staff for all hospital sites.

The study cohort included all individuals who underwent THA or TKA between April 1, 2009, and March 31, 2015 and for whom complete pre-operative data were available. All patients from one hospital were excluded in 2011 because pre-operative questionnaires were not distributed that year.

### Measures

The SF-12 (version 2) is a general-purpose instrument consisting of 12 items that comprise eight sub-domains [21]: physical functioning, role physical, bodily pain, general health, vitality, social functioning, role-emotional, and mental health. The eight sub-domain scores can be weighted and summarized into MH and PH sub-scale scores. According to this model, the items from the physical functioning, role-physical, bodily pain, and general health sub-domains are indicators of PH while vitality, social functioning, role-emotional, and mental health items are indicators for MH. Assessments of construct validity using latent variable models has confirmed this measurement structure [21, 22], although correlations of residual errors for items associated with PH and MH latent variables has been observed [21–23].

Covariates used to describe the study cohort and examine potential DIF sources for the SF-12 included sex, age group, body weight status, and multimorbidity, the presence of two or more chronic conditions [24]. Age was classified as 60 years or less (reference category), 61 to 70 years, and greater than 70 years; the dummy variables AGE1 (0 if age ***≤*** 60 and 1 otherwise) and AGE2 (0 if age ***≤*** 70 and 1 otherwise) were created to represent these age categories. Body weight status was based on body mass index (BMI), which was calculated from measured height and weight (kg/m^2^) captured by clinic nurses; it was categorized as underweight or normal weight (BMI ***≤*** 25*.*0; reference category), overweight (25*.*0 ***<*** BMI ***≤*** 30.0), and obese (BMI > 30*.*0) [25, 26]. The dummy variables of BMI1 (0 if BMI ***≤*** 25.0 and 1 otherwise) and BMI2 (0 if BMI ***≤*** 30.0 and 1 otherwise) were created to represent these categories.

Information about 14 chronic conditions was captured from a self-report questionnaire administered by clinic staff at the pre-operative occasion; individuals were classified as having multimorbidity if they had at least two of these chronic conditions. A single dummy variable COMORB (1 = presence of 2+ comorbid conditions and 0 otherwise) was defined.

### Statistical analysis

The analyses were conducted for patients with complete information (i.e., no missing data) on all SF-12 items. Descriptive analyses were conducted using frequencies and percentages. All analyses were stratified by joint type.

A variety of methods have been used to detect DIF including logistic regression [27], item response theory (IRT) models [28, 29], and the multiple indicators multiple causes (MIMIC) model [30–32]. IRT and MIMIC models can be applied to binary and ordinal item responses, and are flexible to incorporate one or more latent constructs. In addition, the MIMIC is flexible to allow for the specification of dependencies between item residuals [23, 33]. Consequently, we adopted the MIMIC model to test for uniform DIF.

We constructed baseline models for MH and PH sub-scales based on the hypothesized measurement structure of the SF-12, in which the PH and MH items have no cross loading items (Additional file 1: Figures S1 and S2). The baseline models included two correlated residuals (items P2 and P3, P4 and P5) for the PH sub-scale and two correlated residuals (items M1 and M2, M3 and M5) for the MH sub-scale [21, 23] and confirmed by the assessment of fit measures, which demonstrated poorer overall fit when these residuals were not correlated.

**Fig. 1:**
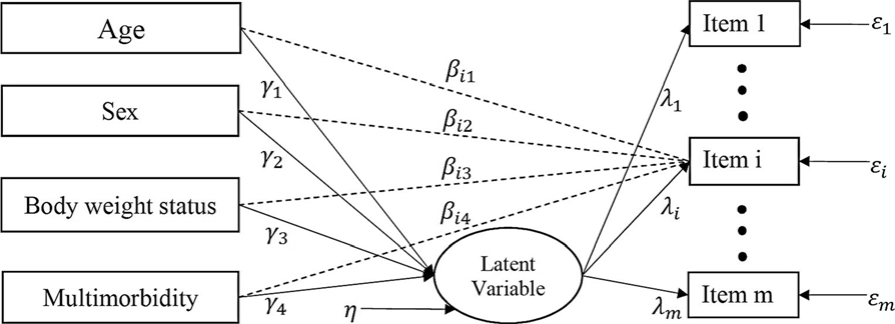
Example of MIMIC model permitting DIF for the *i*th item. The dashed arrow from each covariate to the *i*th item represents the direct effect; *γ*_*k*_ = regression coefficient for the effect of the *k*th covariate on the latent variable; *λ*_*i*_ = the regression coefficient for the latent variable and the *i*th item; *β*_*ik*_ = regression coefficient showing the effect of the *k*th covariate on the *i*th item; *ε*_*i*_ = measurement error for the *i*th item; *η* = residual for the latent variable

In a MIMIC model with *m* items and *k* covariates, the latent response for the *i*th item (*i* = 1,…, *m*) is regressed on the latent variable *F* and the covariate vector **Ζ**,

1 $$ {y}_i^{\ast }=\backslash {\mathrm{uplambda}}_iF+{\boldsymbol{\beta}}_i^{\prime}\mathbf{Z}+\backslash {\mathrm{upvarepsilon}}_i, $$

where *ε*_*i*_ is the error term, *λ*_*i*_ is the factor loading, and ${\boldsymbol{\beta}}_i^{\prime }=\left(\backslash {\mathrm{upbeta}}_{i1}\dots \backslash {\mathrm{upbeta}}_{ik}\right)$ is the vector of the effects of covariates on the latent response ${y}_i^{\ast }$. The latent response is scored via a threshold model

2 $$ {y}_i=c,\kern0.5em if\kern0.5em {\tau}_{i(c)}<{y}_i^{\ast}\le {\tau}_{i\left(c+1\right)}, $$

for categories *c* = 0, 1, 2, …, *C* – 1, where ***τ***_***i***(**0**)_ ***=  − ∞*** and ***τ***_***i***(***C***)_ ***=  + ∞***. Thus, *y*_*i*_ is a polytomous variable which takes discrete values 0, 1, …, *C* – 1. In addition, the latent factor is regressed on the covariates via

3 $$ F=\backslash {\mathrm{upgamma}}^{\prime}\mathbf{Z}+\eta, $$

where *η* is the error term and is independent of **Z**, and **γ**^′^ ***=*** (***γ***_**1**_,  … , ***γ***_***k***_) is a vector of regression coefficients that describe between group differences in *F* (Fig. 1). These formulations enable us to estimate and test ${\boldsymbol{\beta}}_{\boldsymbol{i}}^{\prime }$ conditional on *F*. If ${\boldsymbol{\beta}}_{\boldsymbol{i}}^{\prime}\boldsymbol{\ne}\mathbf{0}$, there is a significant direct effect from the covariates to the latent response ${y}_i^{\ast }$ which means that DIF exists in the *i*th item [34, 35].

There were four primary steps in the DIF analysis. First, unidimensionality of the measurement scales was assessed. Next, anchor items were selected. Then, each item was assessed for DIF. Finally after adjustment for DIF, the contributions of the covariates and items to the final DIF model was assessed.

In the first step the unidimensionality assumption, which implies that all sub-scale items measure a single latent construct, was examined by applying a single-factor model with an oblique rotation to the polychoric correlation matrix for the items for each of the MH and PH sub-scales. To make a decision about unidimensionality, we used two criteria: (a) the existence of only one eigenvalue greater than one, and (b) a large value for the ratio of the first to second eigenvalues (i.e., *r* > 4) [36]. We used several criteria to evaluate the goodness-of-fit of a single-factor model. We considered the model to be a reasonable fit to the data if it had a small root mean square error of approximation (i.e., RMSEA < 0.06), a large comparative fit index (i.e., CFI > 0.95), a large Tucker-Lewis Index (i.e., TLI > 0.95), and a small weighted root mean square residual (i.e., WRMR < 1.0) [37–39].

In the second step, we selected anchor items (i.e., DIF-free items). At least one anchor item must be selected to define the latent construct on which the groups are compared. We used the following method to select the anchor item(s). First, for each sub-scale, a single-factor model was fit to the data; it included direct effects of the covariates on the latent variable but no direct effects between the covariates and the sub-scale items. This was the base model. Next, a series of single-factor models were fit to the data that added direct effects from the covariates; there was one model for each sub-scale item. A *χ*^2^ difference test was used to compare the models with and without the direct effects. The item(s) with the smallest *χ*^2^ statistics was(were) selected as the anchor item(s) [40]. Note that this process was applied to the data for all cohort members so that the same anchor items were selected for both THA and TKA patients. This facilitated the interpretation of the study findings because the same item(s) served as reference points for all analyses. We confirmed the same anchor items in separate analyses for THA and TKA patients.

In the third step, item purification was conducted to identify the items affected by DIF. First, a full model was fit to the data that included direct effects from covariates to all sub-scale items except the anchor item(s). Next, we fit a series of reduced models that excluded direct effects from the covariates to each item; this was done one item at a time. A *χ*^2^ difference test was used to compare these nested models using DIFFTEST for the robust weighted least square estimation method (i.e., WLSMV) in Mplus (https://www.statmodel.com/chidiff.shtml). A large *χ*^2^ difference statistic implies uniform DIF is present for the item.

The fourth step was to fit a model that included direct effects from the covariates to all DIF items (i.e., the items for which DIF was identified in the previous step) and direct effects of the covariates on the latent variable [9, 31]. This model was used to obtain parameter estimates of direct effects of the covariates on the PH and MH sub-scale items. The total effect of DIF was measured via the relative difference between standardized coefficient estimates for the DIF and No-DIF models (i.e., difference in standardized estimates divided by the standardized estimates for the No-DIF model). A difference in standardized coefficients of 0.20 was considered as small, 0.50 as moderate, and 0.80 or greater as large [41]. Estimates of the total effects (i.e., direct and indirect effects) of the covariates on the individual sub-scale items were also produced.

We used an approach based on dominance analysis [42] and Nagelkerke’s coefficient of determination [43–45] to assess the relative importance of both individual items and covariates in the DIF models. Specifically, an item’s importance in the final DIF model was estimated based on its contribution (i.e., direct effects from the covariates to the item) conditional on the contributions of the other items. To measure the item’s importance, a full model was fit to the data that include direct effects of the covariates on all DIF items identified in the previous step, as well as direct effects of the covariates on the latent variable. Next, we fit a series of reduced models that excluded direct effects from the covariates to each DIF item; we did this one item at a time. The importance of each DIF item was assessed using an adaptation of Nagelkerke’s coefficient of determination,

4 $$ {R}^2=\left(1-{e}^{-\left(\Delta {\chi}^2\right)/N}\right)/\left(1-{e}^{-{\chi}_R^2/N}\right), $$

where *N* is the total sample size, ${\chi}_R^2$ is the chi-square test statistic for the reduced model, and Δ*χ*^2^ is the scaled difference in *χ*^2^ test statistics for the reduced and full models. The statistic *R*^2^ is equal to Nagelkerke’s coefficient of determination if we replace ${\chi}_R^2$ with −2 Log(*L*_*R*_) and Δ*χ*^2^ with −2 Log(*L*_*R*_/*L*_*F*_) in maximum likelihood estimation, where *L*_*R*_ and *L*_*F*_ are the likelihood of the reduced and full models, respectively. An item was more important than all other items if it had the largest *R*^2^ amongst all items.

The importance of a covariate in the final DIF model was measured by its contribution (i.e., direct effects from the covariate to all DIF items), conditional on the contribution of the other model covariates. We used a similar approach to that described above to measure covariate importance in the final DIF model. First, a full model was fit to the data that include direct effects from all covariates to the DIF items, as well as direct effects of the covariates on the latent variable. Next, a series of reduced models were fit to the data that excluded the effect of each covariate; this was done one covariate at a time. Using the adapted Nagelkerke coefficient of determination, we measured the importance of each covariate. A covariate was more important than all other covariates if it had the largest *R*^2^ amongst all of the covariates.

All analyses were conducted using Mplus software, version 8. In all analyses, the latent factor mean was constrained to zero and its variance was fixed to one.

## Results

The study cohort included 8820 patients with complete information on all SF-12 items at the pre-operative occasion. Overall, 42.1% patients had THA in the observation period.

For the THA group (Table 1), 53.4% were female, one-third (33.7%) were 60 years of age or younger, and one-third (33.6%) were more than 70 years of age. Overweight and obese individuals accounted for 37.9% and 41.8% of the THA group, respectively. Slightly more than one-half of THA patients had multimorbidity. The most common chronic conditions were hypertension, other (i.e., secondary) osteoarthritis, and back pain.

**Table 1:** Frequency (%) of demographic and health status characteristics of study cohort, stratified by type of joint replacement

Characteristic	THA *n* (%)	TKA *n* (%)	Overall *n* (%)
Total	3714 (42.1)	5106 (57.9)	8820 (100.0)
Sex			
Female	1982 (53.4)	3074 (60.2)	5056 (57.3)
Male	1732 (46.6)	2032 (39.8)	3764 (42.7)
Age			
≤ 60 years	1253 (33.7)	1534 (30.0)	2787 (31.6)
61–70 years	1214 (32.6)	1938 (38.0)	3152 (35.7)
> 70 years	1247 (33.6)	1634 (32.0)	2881 (32.7)
Body Weight Status			
Under weight or normal weight (BMI < = 25.0)	755 (20.3)	533 (10.4)	1288 (14.6)
Overweight (BMI = 25.1–29.9)	1406 (37.9)	1466 (28.7)	2872 (32.6)
Obese (BMI = 30.0+)	1553 (41.8)	3107 (60.8)	4660 (52.8)
Multimorbidity			
2+ Chronic conditions	1986 (53.5)	3174 (62.2)	5160 (58.5)
< 2 Chronic conditions	1728 (46.5)	1932 (37.8)	3660 (41.5)
Chronic Conditions			
Hypertension	1639 (44.1)	2725 (53.4)	4364 (49.5)
Other osteoarthritis	1476 (39.7)	2272 (44.5)	3748 (42.5)
Back pain	1450 (39.0)	1914 (37.5)	3364 (38.1)
Diabetes	417 (11.2)	913 (17.9)	1330 (15.1)
Heart disease	385 (10.4)	559 (10.9)	944 (10.7)
Depression	395 (10.6)	705 (13.8)	1100 (12.5)
Rheumatoid arthritis	294 (7.9)	585 (11.5)	879 (10.0)
Stomach ulcer	168 (4.5)	337 (6.6)	505 (5.7)
Cancer	183 (4.9)	234 (4.6)	417 (4.7)
Anemia	153 (4.1)	228 (4.5)	381 (4.3)
Lung disease	165 (4.4)	263 (5.2)	428 (4.9)
Kidney disease	72 (1.9)	104 (2.0)	176 (2.0)
Liver disease	36 (1.0)	58 (1.1)	94 (1.1)
Other condition	741 (20.0)	1165 (22.8)	1906 (21.6)

*THA* Total hip arthroplasty, *TKA* Total knee arthroplasty, *BMI* Body mass index

For the TKA group, 60.2% were female and approximately one-third (30.0%) were 60 years of age or younger and one-third (32.0%) were more than 70 years of age. Overweight and obese individuals accounted for 28.7% and 60.8% of patients, respectively. Multimorbidity was identified in almost two-thirds of TKA patients (62.2%) and the most common chronic conditions were the same as for the THA group.

The frequencies of responses to the MH and PH sub-scale items are reported in Table 2 for the entire cohort. For the MH sub-scale, close to half (46.4%) of patients responded “A little of the time” to M3 (“felt calm and peaceful”), while 36.0% of patients responded “None of the time” to M2 (“less careful than usual”). Furthermore, for the PH sub-scale, more than half (58.9%) of patients respond “Yes, limited a lot” to P2 (“moderate activities”) while 74.4% of patients respond “Yes, limited a lot” to P3 (“climbing several flights of stairs”).

**Table 2:** Frequencies (%) of responses to the SF-12 mental health (MH) and physical health (PH) sub-scale items

MH Sub-scale					
	All of the time	Most of the time	Some of the time	A little of the time	None of the time
M1: Accomplished less than would like	634 (7.2)	1324 (15.0)	2108 (23.9)	1858 (21.1)	2896 (32.8)
M2: Less careful than usual	564 (6.4)	1240 (14.1)	1998 (22.7)	1841 (20.9)	3177 (36.0)
M3: Felt calm and peaceful	373 (4.2)	1227 (13.9)	2510 (28.5)	4094 (46.4)	616 (7.0)
M4: Have a lot of energy	1017 (11.5)	2401 (27.2)	3150 (35.7)	2027 (23.0)	225 (2.6)
M5: Felt downhearted and depressed	165 (1.9)	595 (6.7)	2254 (25.6)	2860 (32.4)	2946 (33.4)
	Not at all	A little bit	Moderately	Quite a bit	Extremely
M6: Have social limitations	556 (6.3)	1352 (15.3)	2545 (28.9)	1915 (21.7)	2452 (27.8)
PH Sub-scale					
	Excellent	Very good	Good	Fair	Poor
P1: General health	150 (1.7)	1021 (11.6)	4181 (47.4)	2892 (32.8)	576 (6.5)
	Yes, limited a lot	Yes, limited a little	No, not limited at all		
P2: Limits in moderate activity	5198 (58.9)	3031 (34.4)	591 (6.7)		
P3: Climbing several flights of stairs	6563 (74.4)	1910 (21.7)	347 (3.9)		
	All of the time	Most of the time	Some of the time	A little of the time	None of the time
P4: Accomplished less than would like	2564 (29.1)	3199 (36.3)	2049 (23.2)	731 (8.3)	277 (3.1)
P5: Limited in work and activity	2680 (30.4)	3291 (37.3)	1989 (22.6)	633 (7.2)	227 (2.6)
P6: Have pain with normal work	1825 (20.7)	3942 (44.7)	2118 (24.0)	804 (9.1)	131 (1.5)

With respect to multimorbidity, 19.0% of individuals in the cohort had no chronic conditions and 22.5% had a single chronic condition. Almost one-quarter (24.2%) had two chronic conditions, and the remainder had three or more chronic conditions.

Exploratory factor analysis revealed that for both the PH and MH sub-scales there existed only one eigenvalue with a value greater than one. The ratio of the first to second eigenvalues was larger than four, except for the PH sub-scale in the TKA group where it was only slightly less than this criterion (*r* = 3.95). In addition, the second eigenvalue was similar in size to the third eigenvalue in both groups and for both sub-scales. Therefore, it was reasonable to accept unidimensionality of the MH and PH sub-scales for both the THA and TKA groups.

In the baseline model for the PH and MH sub-scales, two correlated residuals for the PH sub-scale (items P2 and P3, P4 and P5) and four correlated residuals for the MH sub-scale (items M1 and M2, M3 and M5) were considered for inclusion based on the empirical results and previous research [21, 23]. Adding residual correlations for these items resulted in an acceptable model fit (Table 3). Specifically, the single-factor model fit to the MH sub-scale items for the THA group had RMSEA = 0.05, CFI = 1.00, TLI = 1.00, and WRMR = 0.62 and for the TKA group it had RMSEA = 0.03, CFI = 1.00, TLI = 1.00, and WRMR = 0.38 for the TKA group. The single-factor model fit to the PH sub-scale items had RMSEA = 0.03, CFI = 1.00, TLI = 1.00, and WRMR = 0.61 for the THA group. For the TKA group, this model had RMSEA = 0.02, CFI = 1.00, TLI = 1.00, and WRMR = 0.52.

**Table 3:** Goodness-of-fit statistics for the SF-12 mental health (MH) and physical health (PH) sub-scales with and without correlated residual variances, stratified by type of joint replacement

Model	Joint Type	RMSEA (90% CI)	CFI	TLI	WRMR
MH Sub-scale					
No residual variance correlations	THA	0.22 (0.21, 0.23)	0.97	0.94	4.77
	TKA	0.21 (0.21, 0.22)	0.97	0.95	5.18
With residual variance correlations: items M1 and M2, M3 and M5	THA	0.15 (0.14, 0.15)	0.99	0.97	2.47
	TKA	0.14 (0.13, 0.15)	0.99	0.98	2.76
With residual variance correlations: items M1 and M2, M3 and M5, M3 and M4, M4 and M6	THA	**0.05 (0.04, 0.07)**	**1.00**	**1.00**	**0.62**
	TKA	**0.03 (0.02, 0.04)**	**1.00**	**1.00**	**0.38**
PH Sub-scale					
No residual variance correlations	THA	0.19 (0.18, 0.20)	0.98	0.97	4.49
	TKA	0.15 (0.14, 0.15)	0.98	0.97	3.95
With residual correlations: items P2 and P3, P4 and P5	THA	**0.03 (0.02, 0.04)**	**1.00**	**1.00**	**0.61**
	TKA	**0.02 (0.01, 0.03)**	**1.00**	**1.00**	**0.52**

*THA* Total hip arthroplasty, *TKA* Total knee arthroplasty, *RMSEA* Root mean square error of approximation, *CFI* Comparative fit index, *TLI* Tucker-Lewis index, *WRMR* Weighted root mean square residual, *CI* Confidence interval; values in boldface font represent the fit statistics for the best-fitting models

We selected anchor items empirically for each sub-scale. We selected items M5 (“Felt downhearted and depressed”) and M6 (“Social limitations”) for the MH sub-scale and items P2 (“Moderate activity”) and P6 (“Have pain with normal work)” for the PH sub-scale, because they had the smallest *χ*^2^ statistics. Specifically, the *χ*^2^ statistics had values of 96.9, 157.1, 150.9, 160.1, 17.7, and 26.4 for items M1 to M6, respectively and values of 719.6, 41.0, 136.9, 62.2, 99.2, and 43.5 for items P1 to P6, respectively. .

Then we tested all non-anchor items for uniform DIF. For the MH sub-scale, all of the *χ*^2^ difference tests produced statistically significant results for both the TKA and THA groups, suggesting uniform DIF was present in all non-anchor items (see Table 4). Furthermore, the χ^2^ difference tests suggested the presence of uniform DIF in all non-anchor items for the PH sub-scale for both the THA and TKA groups.

**Table 4:** Tests for differential item functioning on the SF-12 mental health (MH) and physical health (PH) sub-scale items, stratified by type of joint replacement

Item	THA			TKA		
	Δχ^2^	df	*p*-value	Δχ2	df	*p*-value
MH Sub-scale						
M1	20.09	6	0.003	37.03	6	< 0.001
M2	31.85	6	< 0.001	73.22	6	< 0.001
M3	38.30	6	< 0.001	44.61	6	< 0.001
M4	99.26	6	< 0.001	83.93	6	< 0.001
M5	*	*	*	*	*	*
M6	*	*	*	*	*	*
PH Sub-scale						
P1	244.32	6	< 0.001	425.16	6	< 0.001
P2	*	*	*	*	*	*
P3	27.97	6	< 0.001	53.76	6	< 0.001
P4	12.96	6	0.044	16.34	6	0.012
P5	19.78	6	0.003	20.25	6	0.003
P6	*	*	*	*	*	*

*df* Degree of freedom, Δχ^2^ = Chi-square difference test obtained using the DIFFTEST procedure for WLSMV in MPlus; * = anchor item; All tests are statistically significant at α = 0.05

Table 5 provides estimates of the direct effects of the covariates on the latent variables. As well, differences in the estimates when there were direct effects from the covariates to the items (i.e., DIF model) versus the case when there were no direct effects from covariates to the items (i.e., No-DIF model) are provided. As Table 5 reveals, in both the THA and TKA groups, the PH and MH latent variables were always negatively associated with the covariates COMORB and BMI2 and positively associated with sex. This indicates that patients with multimorbidity had smaller PH and MH latent variable scores on average, relative to other patients; obese patients had smaller PH and MH latent variable scores relative to non-obese patients, and the PH and MH latent variable scores for men were always larger than those for women. Almost all of the estimates were statistically significant in both the DIF and No-DIF models. The relative differences revealed that the largest effects of the covariates on the MH latent variable were observed for the age and body weight status covariates in the THA group. The majority of the standardized differences indicate small effects; the exceptions were for the covariates AGE2 and BMI1 for the THA group, which were moderate in size. For the PH latent variable, all of the covariates had smaller relative difference statistics than for the MH latent variable in both the TKA and THA groups.

**Table 5:** Regression model estimates for covariate associations with SF-12 mental health (MH) and physical health (PH) latent variable scores

Covariate	MH Latent Variable					PH Latent Variable				
	No-DIF Model		DIF Model		d	No-DIF Model		DIF Model		d
	Est	SE	Est	SE		Est	SE	Est	SE	
THA										
AGE1	**0.17**	0.05	**0.21**	0.05	0.10	0.08	0.05	0.08	0.05	0.00
AGE2	0.08	0.05	**0.13**	0.05	0.50	**−0.12**	0.05	**−0.14**	0.05	0.11
BMI1	0.07	0.05	**0.11**	0.06	0.43	−0.06	0.05	−0.06	0.05	−0.07
BMI2	**−0.20**	0.04	**−0.21**	0.05	−0.03	**− 0.25**	0.04	**− 0.20**	0.04	− 0.20
SEX	**0.32**	0.04	**0.28**	0.04	− 0.21	**0.32**	0.04	**0.32**	0.04	−0.04
COMORB	**−0.53**	0.04	**−0.52**	0.04	−0.13	**− 0.37**	0.04	**− 0.33**	0.04	− 0.10
TKA										
AGE1	**0.27**	0.04	**0.28**	0.04	0.10	**0.27**	0.04	**0.24**	0.04	−0.11
AGE2	**0.33**	0.05	**0.28**	0.04	−0.09	**0.22**	0.04	**0.19**	0.04	−0.19
BMI1	**0.20**	0.06	**0.15**	0.06	−0.15	**0.12**	0.06	**0.13**	0.06	0.07
BMI2	**−0.15**	0.04	**−0.15**	0.04	0.12	**−0.29**	0.04	**−0.27**	0.04	−0.10
SEX	**0.30**	0.04	**0.30**	0.03	0.09	**0.24**	0.03	**0.22**	0.03	−0.12
COMORB	**−0.50**	0.04	**−0.49**	0.03	0.06	**−0.37**	0.03	**−0.32**	0.03	−0.13

AGE1 = 0 if age ≤ 60 and 1 otherwise; AGE2 = 0 if age ≤ 70 and 1 otherwise; BMI1 = 0 if BMI ≤ 25.0 and 1 otherwise); BMI2 = 0 if BMI ≤ 30.0 and 1 otherwise; COMORB = 0 if < 2 comorbid conditions and 1 otherwise; *EST* Estimate, *SE* Standard error, *d* Relative difference in standardized estimates between DIF model and No-DIF models; Boldface font is used to denote statistically significant estimates at α = 0.05

Adjustment for DIF resulted in changes in the estimates of the total effects for most of the SF-12 MH and PH sub-scale items (Additional file 2: Tables S1 and S2). For the MH sub-scale items, in general the largest relative differences in total effect estimates for the DIF and no-DIF models were associated with the age and body weight status covariates; these differences were generally larger in size for the THA group than for the TKA group. For the PH sub-scale items, the relative differences in total effect estimates for the DIF and no-DIF models were all small, except for item P1 (“General health”) in the THA group.

**Fig. 2:**
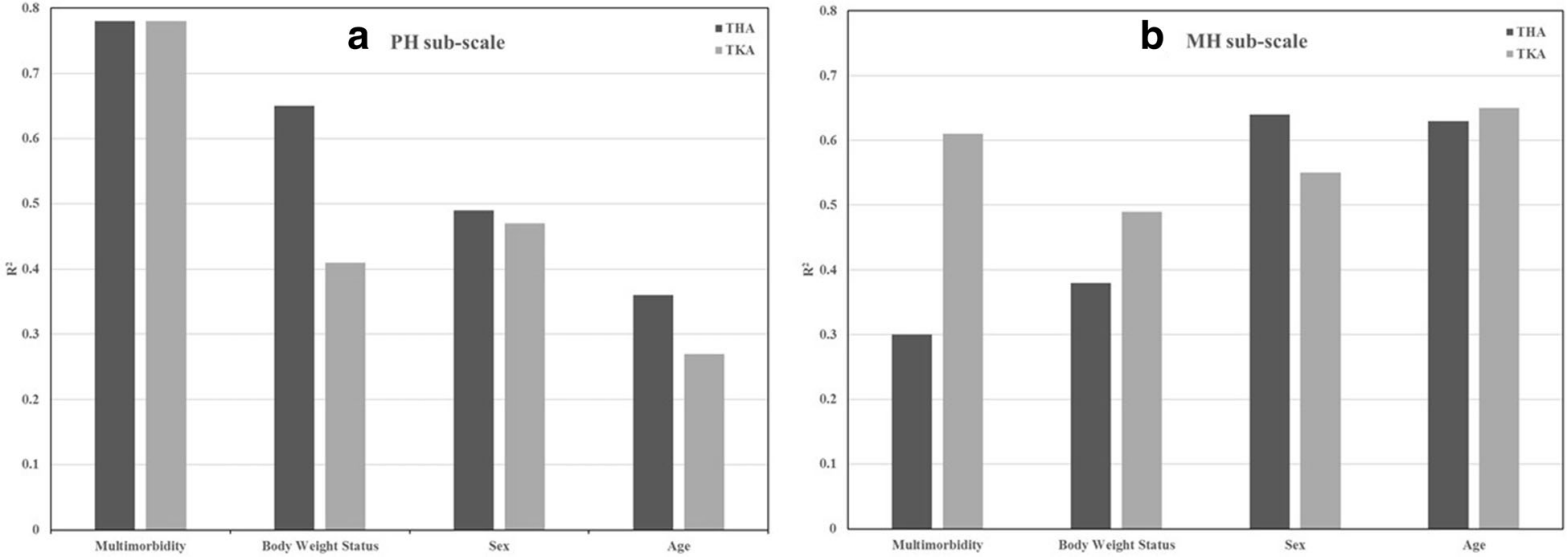
Importance of the covariates in the final DIF models. **a** Importance analysis for the PH sub-scale. **b** Importance analysis for the MH sub-scale

In the final DIF model for the PH sub-scale, multimorbidity and age had the largest and smallest relative importance, respectively as judged by the modified coefficient of determination (Fig. 2). This finding was consistent for both the THA and TKA groups. In the final DIF model for the MH sub-scale, sex and age had the largest importance for the THA group while multimorbidity and age had the largest *R*^2^ statistics for the TKA group.

**Fig. 3:**
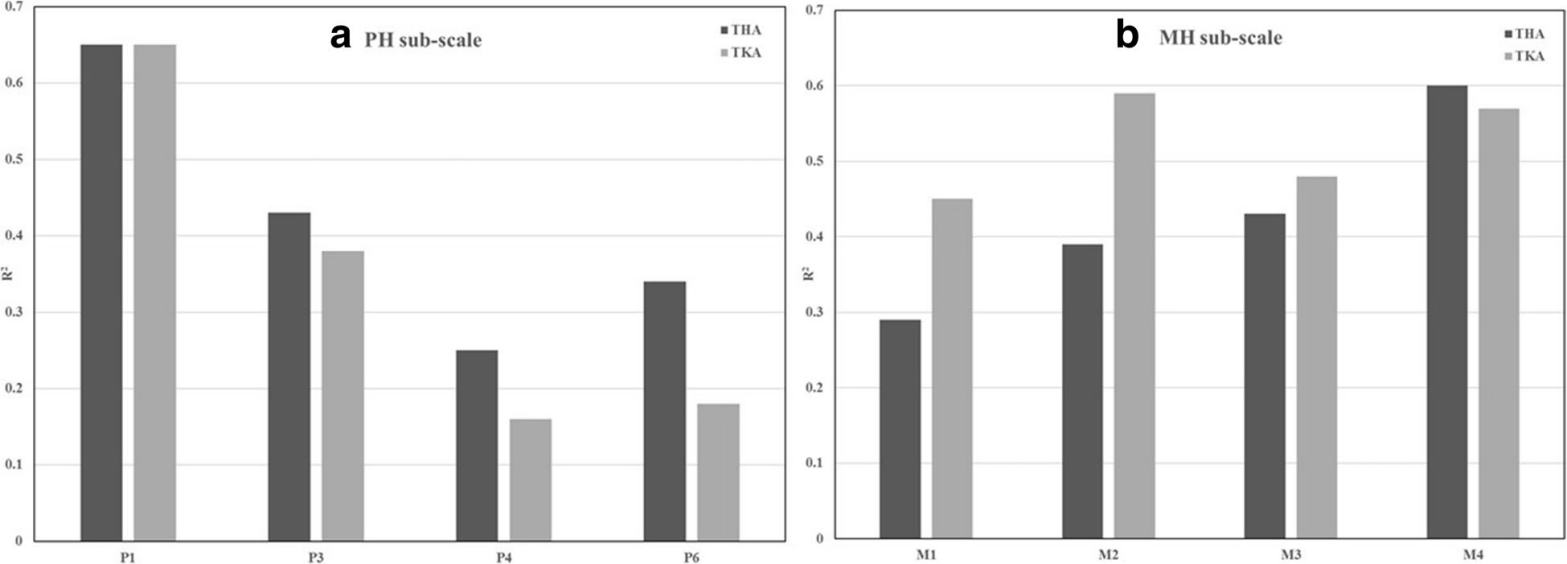
Importance of the items in the final DIF models. **a** Importance of the PH sub-scale items. **b** Importance of the MH sub-scale items

Finally, the relative importance analyses were conducted for all sub-scale items (Fig. 3). For the PH sub-scale, item P1 (“General health”) had the largest contribution to the final DIF model while item P4 (“Accomplished less, physical”) had the smallest contribution. For the MH sub-scale, item M4 (“Have a lot of energy”) for the THA group and items M4 and M2 (“Less careful than usual”) for the TKA group had the largest contributions to the final DIF model.

## Discussion

This study tested for DIF in the PH and MH sub-scale items of the SF-12 across demographic and health status characteristics for patients having joint replacement surgery. We focused on responses given prior to surgery, as this is when health status measures (i.e., body weight status and presence of comorbid conditions) were collected, and also because pre-surgery assessments are an essential reference point for assessing the magnitude of post-surgery improvements [46]. The responses given by patients on the SF-12 items have some consistency with previous research, which has shown, for example, that older patients are more likely to report problems with moderate activities and climbing several flights of stairs [47, 48].

Our findings suggest that multimorbidity is not only a source of DIF but also had the largest contribution to the DIF model for the PH sub-scale in the relative importance analysis. Other recent studies have shown a strong association between SF-12 PH sub-scale scores and multimorbidity [49], although this covariate has not been explored for its effect in DIF analyses. At the same time, the differences in estimates of the effect of comorbidity on the MH and PH latent variables between the No-DIF and DIF models were generally small.

Item M4 from the MH sub-scale and item P1 from the PH sub-scale were associated with the largest contributions to the final DIF models. Adjustment for DIF did not change the direction of the association between the covariates and the PH and MH scores. This result was consistent with other findings in the literature for the SF-12 and also for the SF-36 [9, 14].

While this study investigated DIF in a population for which PROMs are of significant value for assessing surgical outcomes, further research is warranted. Since DIF may change from pre-surgery to post-surgery occasions, future studies might explore response shift [50], a change in an individual’s values, internal standards, and conceptualization of QOL over time, in joint replacement populations. Research conducted to date [51–53] has identified the presence of response shift in patients undergoing total knee replacement. As well, we only tested for uniform DIF in MH and PH. The MIMIC model cannot easily be used to investigate the presence of non-uniform DIF, which involves testing interactions between covariates and latent variables on the item responses. Specification of interaction terms assumes normally distributed covariates [32]. Thus, there is opportunity for opportunities to investigate new approaches to test for non-uniform DIF in MIMIC models. Finally, the generalizability of the study findings regarding the measurement model fit to the data and presence of DIF should be explored in other joint replacement populations.

## Conclusions

In summary, this study suggests the existence of DIF in population-based SF-12 data for joint replacement patients. PH and MH sub-scale scores may not be comparable across sub-groups defined by demographic and health status variables without considering the effects of DIF. Moreover, this study has provided evidence that having more than one chronic condition may be a source of DIF; multimorbidity should therefore be explored further in studies about DIF in other populations. At the same time, associations between the latent construct and the covariates revealed generally small differences between the DIF and no-DIF models, indicating that the effect of DIF on the latent construct was not substantial in either THA or TKA patients.

DIF should be given routine consideration in the analysis of PROMs because it can impact the interpretation of group differences. Measurement equivalence is essential to ensure accurate assessments of patient health; inaccurate assessment can result in incorrect estimates of the magnitude of group differences and can impact on clinical decision making about the effectiveness of interventions, such as THA and TKA, on patient’s perceptions of their own health.

There are a few methods to address the presence of DIF in PROMs data, although no method is recognized as the optimal solution [54]. Removing DIF items from the SF-12 is likely to effect the validity and accuracy of this measure. Replacing DIF items with equivalent items that do not exhibit DIF is conditional on having a resource of known DIF-free items. Examining items for DIF prior to conducting analyses on the SF-12 and adjusting for DIF before comparing sub-groups may be a reasonable solution, although it can also affect the comparability of scores across populations. Sensitivity analyses, in which analyses of PROMs are conducted after accounting for DIF and then not accounting for DIF, is a feasible approach for researchers to adopt in practice.

## Additional files

Additional file 1: **Figure S1.** Baseline model for the SF-12 mental health sub-scale. **Figure S2.** Baseline model for the SF-12 physical health sub-scale. (PDF 100 kb)

Additional file 2: **Table S1.** Total effects of covariates on the SF-12 mental health sub-scale items for differential item functioning (DIF) and No-DIF models, stratified by type of joint replacement. **Table S2.** Total effects of covariates on the SF-12 physical health sub-scale items for differential item functioning (DIF) and No-DIF models, stratified by type of joint replacement. (PDF 189 kb)

## Data Availability

Data used in this article were derived from administrative health data as a secondary source. The data were provided under specific data sharing agreements only for the approved use. The original source data are not owned by the researchers and as such cannot be provided to a public repository. The original data source and approval for use has been noted in the acknowledgments of the article. Where necessary and with appropriate approvals, source data specific to this article or project may be reviewed with the consent of the original data providers, along with the required privacy and ethical review bodies.

## Abbreviations

BMI: Body mass index
COMORB: Comorbidity
DIF: Differential item functioning
HRQoL: Health-related quality of life
MH: Mental health
PH: Physical health
PROM: Patient-reported outcome measure
THA: Total hip arthroplasty
TKA: Total knee arthroplasty
