# Supplementary material for: Differential item functioning of the SF-12 in a population-based regional joint replacement registry
Source: Health Qual Life Outcomes. 2019 Jul 2;17:114. doi: 10.1186/s12955-019-1166-1 (PMC6604189; doi:10.1186/s12955-019-1166-1)
Supplement: Supplementary file 1 — Figure S1. Baseline model for the SF-12 mental health sub-scale. Figure S2. Baseline model for the SF-12 physical health sub-scale. (PDF 100 kb) [file 12955_2019_1166_MOESM1_ESM.pdf]

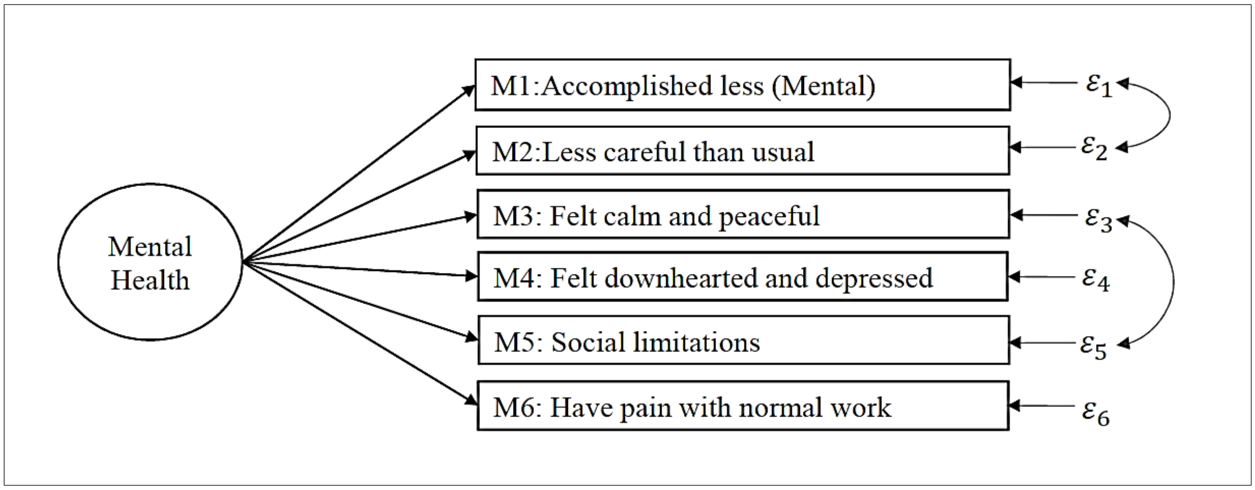

Figure S1. Baseline model for the SF-12 mental health sub-scale. M1-Role Functioning (Emotional): accomplished less (emotional problems); M2-Role Functioning (Emotional): less carefully than usual; M3-Mental Health: calm and peaceful; M4-Mental Health: downhearted and depressed; M5-Social Functioning: social limitations; M6-Bodily Pain: have pain with normal work

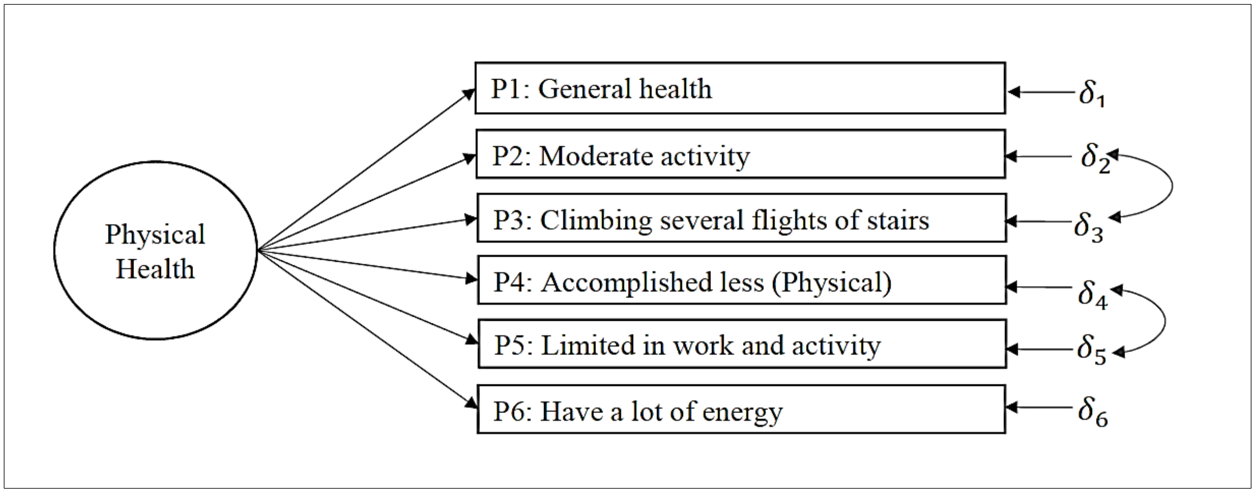

Figure S2. Baseline model for the SF-12 physical health sub-scale. P1-General Health: rating of general health; P2-Physical Functioning: moderate activities; P3-Physical Functioning: climbing several flights of stairs; P4-Role Functioning (Physical): accomplished less; P5-Role Functioning (Physical): limited in work or other activities; P6-Vitality: energy
