# Supplementary material for: Differential item functioning of the SF-12 in a population-based regional joint replacement registry
Source: Health Qual Life Outcomes. 2019 Jul 2;17:114. doi: 10.1186/s12955-019-1166-1 (PMC6604189; doi:10.1186/s12955-019-1166-1)
Supplement: Supplementary file 2 — Table S1. Total effects of covariates on the SF-12 mental health sub-scale items for differential item functioning (DIF) and No-DIF models, stratified by type of joint replacement. Table S2. Total effects of covariates on the SF-12 physical health sub-scale items for differential item functioning (DIF) and No-DIF models, stratified by type of joint replacement. (PDF 189 kb) [file 12955_2019_1166_MOESM2_ESM.pdf]

Table S1. Total effects of covariates on the SF-12 mental health sub-scale items for differential item functioning (DIF) and No-DIF models, stratified by type of joint replacement

		THA					TKA				
		No-DIF Model		DIF Model			No-DIF Model		DIF Model		
**Item**	**Covariate**	**Est**	**SE**	**Est**	**SE**	**d**	**Est**	**SE**	**Est**	**SE**	**d**
M1	AGE1	**0.16**	0.04	**0.14**	0.05	-0.30	**0.26**	0.04	**0.22**	0.04	-0.15
M1	AGE2	0.08	0.04	0.01	0.05	-0.90	**0.26**	0.04	**0.19**	0.05	-0.42
M1	BMI1	0.07	0.05	0.02	0.06	-0.76	**0.14**	0.06	0.11	0.06	-0.21
M1	BMI2	**-0.19**	0.04	**-0.14**	0.05	-0.41	**-0.15**	0.04	**-0.13**	0.04	-0.13
M1	SEX	**0.29**	0.04	**0.19**	0.04	-0.34	**0.28**	0.03	**0.18**	0.04	-0.52
M1	COMORB	**-0.53**	0.04	**-0.48**	0.05	-0.28	**-0.49**	0.03	**-0.41**	0.04	-0.37
M2	AGE1	**0.14**	0.04	0.05	0.05	-0.71	**0.22**	0.03	**0.13**	0.04	-0.56
M2	AGE2	0.07	0.04	-0.10	0.05	-2.14	**0.22**	0.03	0.02	0.04	-0.93
M2	BMI1	0.06	0.04	0.04	0.06	-0.56	**0.12**	0.05	0.10	0.06	-0.31
M2	BMI2	**-0.17**	0.04	**-0.17**	0.05	-0.20	**-0.13**	0.03	**-0.13**	0.04	-0.25
M2	SEX	**0.25**	0.03	**0.19**	0.04	-0.43	**0.24**	0.03	**0.18**	0.03	-0.25
M2	COMORB	**-0.47**	0.04	**-0.42**	0.04	-0.11	**-0.43**	0.03	**-0.31**	0.04	-0.46
M3	AGE1	**0.11**	0.03	**0.20**	0.04	0.36	**0.16**	0.02	**0.23**	0.03	-0.04
M3	AGE2	0.05	0.03	**0.26**	0.04	2.90	**0.16**	0.02	**0.31**	0.03	0.29
M3	BMI1	0.05	0.03	0.09	0.05	0.08	**0.09**	0.03	0.08	0.05	-0.47
M3	BMI2	**-0.13**	0.03	**-0.08**	0.04	-0.54	**-0.09**	0.02	-0.02	0.03	-0.85
M3	SEX	**0.20**	0.02	**0.25**	0.03	-0.17	**0.17**	0.02	**0.25**	0.03	-0.02
M3	COMORB	**-0.35**	0.03	**-0.38**	0.04	-0.19	**-0.29**	0.02	**-0.32**	0.03	-0.26
M4	AGE1	**0.08**	0.02	0.02	0.04	-0.88	**0.12**	0.02	**0.20**	0.03	0.11
M4	AGE2	0.04	0.02	**-0.10**	0.04	-2.25	**0.12**	0.02	**0.14**	0.04	-0.42
M4	BMI1	0.03	0.02	-0.02	0.05	-1.27	**0.07**	0.03	0.06	0.05	-0.49
M4	BMI2	**-0.09**	0.02	**-0.21**	0.04	0.17	**-0.07**	0.02	**-0.21**	0.03	1.00
M4	SEX	**0.14**	0.02	**0.37**	0.03	0.76	**0.13**	0.01	**0.22**	0.03	-0.44
M4	COMORB	**-0.24**	0.02	**-0.34**	0.03	-0.06	**-0.21**	0.02	**-0.39**	0.03	0.24
M5	AGE1	**0.12**	0.03	**0.15**	0.04	-0.06	**0.20**	0.03	**0.20**	0.03	0.00
M5	AGE2	0.06	0.03	**0.09**	0.04	0.13	**0.21**	0.03	**0.24**	0.03	0.14
M5	BMI1	0.05	0.04	0.08	0.04	0.60	**0.11**	0.04	**0.15**	0.05	0.09
M5	BMI2	**-0.15**	0.03	**-0.15**	0.04	-0.25	**-0.12**	0.03	**-0.11**	0.03	-0.08
M5	SEX	**0.22**	0.03	**0.19**	0.03	-0.14	**0.22**	0.02	**0.22**	0.03	-0.33
M5	COMORB	**-0.40**	0.03	**-0.39**	0.03	-0.02	**-0.39**	0.03	**-0.40**	0.03	0.03

M6	AGE1	**0.13**	0.04	**0.16**	0.04	0.23	**0.21**	0.03	**0.21**	0.03	0.00
M6	AGE2	0.06	0.04	**0.10**	0.04	0.67	**0.21**	0.03	**0.25**	0.04	-0.11
M6	BMI1	0.06	0.04	0.09	0.04	0.50	**0.12**	0.05	**0.15**	0.05	0.25
M6	BMI2	**-0.15**	0.03	**-0.16**	0.04	-0.20	**-0.12**	0.03	**-0.12**	0.03	0.00
M6	SEX	**0.24**	0.03	**0.21**	0.03	-0.13	**0.23**	0.03	**0.23**	0.03	0.00
M6	COMORB	**-0.41**	0.03	**-0.40**	0.03	-0.02	**-0.39**	0.03	**-0.40**	0.03	0.03

Note: AGE1 = 0 if age ≤ 60 and 1 otherwise; AGE2 = 0 if age ≤ 70 and 1 otherwise; BMI1= 0 if BMI ≤ 25.0 and 1 otherwise; BMI2 = 0 if BMI ≤ 30.0 and 1 otherwise; COMORB = 0 if < 2 comorbid conditions and 1 otherwise; EST=estimate; SE= standard error; d=relative difference in standardized estimates between DIF model and No-DIF models; Boldface font is used to denote statistically significant estimates at α=0.05.

Table S2. Total effects of covariates on the SF-12 physical health sub-scale items for differential item functioning (DIF) and No-DIF models, stratified by type of joint replacement

		THA					TKA				
		No-DIF Model		DIF Model			No-DIF Model		DIF Model		
Item	Covariate	Est	SE	Est	SE	d	Est	SE	Est	SE	d
P1	AGE1	0.01	0.01	**0.08**	0.03	1.67	**0.05**	0.01	**0.11**	0.03	-0.27
P1	AGE2	**-0.02**	0.01	**-0.08**	0.03	0.33	**0.04**	0.01	**0.09**	0.03	-0.25
P1	BMI1	-0.01	0.01	0.00	0.04	-1.00	**0.02**	0.01	-0.02	0.04	-1.25
P1	BMI2	**-0.04**	0.01	**-0.26**	0.03	1.17	**-0.05**	0.01	**-0.19**	0.03	0.27
P1	SEX	**0.05**	0.01	0.02	0.03	-0.87	**0.04**	0.01	0.04	0.02	-0.50
P1	COMORB	**-0.06**	0.01	**-0.45**	0.03	1.50	**-0.06**	0.01	**-0.51**	0.02	3.25
P2	AGE1	0.03	0.02	0.04	0.02	0.33	**0.11**	0.02	**0.11**	0.02	0.00
P2	AGE2	**-0.04**	0.02	**-0.05**	0.02	0.25	**0.09**	0.02	**0.08**	0.02	-0.11
P2	BMI1	-0.02	0.02	-0.02	0.02	0.00	**0.05**	0.02	0.04	0.03	-0.47
P2	BMI2	**-0.09**	0.02	**-0.07**	0.02	-0.22	**-0.11**	0.01	**-0.09**	0.02	-0.59
P2	SEX	**0.13**	0.02	**0.15**	0.02	0.15	**0.10**	0.01	**0.10**	0.02	-0.50
P2	COMORB	**-0.12**	0.01	**-0.12**	0.02	-0.50	**-0.14**	0.01	**-0.13**	0.02	-0.54
P3	AGE1	0.03	0.02	0.00	0.03	-1.00	**0.06**	0.01	**0.06**	0.02	-0.50
P3	AGE2	**-0.04**	0.02	**-0.06**	0.03	0.00	**0.05**	0.01	0.03	0.02	-0.70
P3	BMI1	-0.02	0.02	-0.03	0.03	0.00	**0.03**	0.01	0.00	0.03	-1.00
P3	BMI2	**-0.07**	0.01	**-0.10**	0.02	-0.29	**-0.06**	0.01	**-0.07**	0.02	-0.42
P3	SEX	**0.11**	0.01	**0.21**	0.03	-0.36	**0.06**	0.01	**0.18**	0.02	0.50
P3	COMORB	**-0.11**	0.01	**-0.10**	0.02	-0.55	**-0.07**	0.01	**-0.07**	0.01	0.00
P4	AGE1	0.07	0.04	0.06	0.04	-0.14	**0.22**	0.03	**0.16**	0.04	-0.45
P4	AGE2	**-0.11**	0.04	**-0.13**	0.05	-0.05	**0.18**	0.03	**0.14**	0.04	-0.42
P4	BMI1	-0.05	0.05	-0.05	0.05	0.00	**0.10**	0.05	**0.14**	0.05	0.40
P4	BMI2	**-0.21**	0.04	**-0.19**	0.04	-0.10	**-0.23**	0.03	**-0.24**	0.03	0.04
P4	SEX	**0.30**	0.04	**0.23**	0.04	-0.23	**0.20**	0.03	**0.14**	0.03	-0.30
P4	COMORB	**-0.31**	0.03	**-0.26**	0.04	-0.37	**-0.29**	0.03	**-0.24**	0.03	-0.17
P5	AGE1	0.07	0.04	0.07	0.04	0.00	**0.22**	0.03	**0.23**	0.03	0.05
P5	AGE2	**-0.10**	0.04	-0.03	0.04	-0.70	**0.18**	0.03	**0.26**	0.04	0.08
P5	BMI1	-0.05	0.04	-0.05	0.05	-0.20	**0.10**	0.05	**0.13**	0.05	0.30
P5	BMI2	**-0.20**	0.03	**-0.19**	0.04	-0.29	**-0.22**	0.03	**-0.19**	0.03	-0.14
P5	SEX	**0.28**	0.03	**0.21**	0.04	-0.44	**0.19**	0.03	**0.13**	0.03	-0.32
P5	COMORB	**-0.29**	0.03	**-0.25**	0.03	-0.14	**-0.28**	0.03	**-0.22**	0.03	-0.21

P6	AGE1	0.06	0.03	0.07	0.04	-0.13	**0.17**	0.03	**0.18**	0.03	0.06
P6	AGE2	**-0.09**	0.03	**-0.09**	0.04	-0.25	**0.14**	0.03	**0.13**	0.03	-0.07
P6	BMI1	-0.04	0.04	-0.04	0.04	0.00	**0.08**	0.04	0.06	0.04	-0.25
P6	BMI2	**-0.17**	0.03	**-0.13**	0.03	-0.24	**-0.18**	0.02	**-0.15**	0.03	-0.44
P6	SEX	**0.24**	0.03	**0.27**	0.03	0.13	**0.15**	0.02	**0.16**	0.02	0.07
P6	COMORB	**-0.25**	0.03	**-0.23**	0.03	-0.08	**-0.23**	0.02	**-0.21**	0.02	-0.09

Note: AGE1 = 0 if age ≤ 60 and 1 otherwise; AGE2 = 0 if age ≤ 70 and 1 otherwise; BMI1= 0 if BMI ≤ 25.0 and 1 otherwise; BMI2 = 0 if BMI ≤ 30.0 and 1 otherwise; COMORB = 0 if < 2 comorbid conditions and 1 otherwise; EST=estimate; SE= standard error; d=relative difference in standardized estimates between DIF model and No-DIF models; Boldface font is used to denote statistically significant estimates at α=0.05.
